# Inverted ILM Flap for the Treatment of Myopic Macular Holes: Healing Processes and Morphological Changes in Comparison with Complete ILM Removal

**DOI:** 10.1155/2019/1314989

**Published:** 2019-06-02

**Authors:** Maurizio Mete, Alessandro Alfano, Emilia Maggio, Massimo Guerriero, Grazia Pertile

**Affiliations:** ^1^Department of Ophthalmology, Sacro Cuore-Don Calabria Hospital, Negrar, Verona, Italy; ^2^Department of Computer Science, University of Verona, Verona, Italy

## Abstract

**Purpose:**

To investigate the microstructural changes after successful myopic macular hole (MMH) surgery, comparing inverted ILM flap and complete ILM removal techniques, and their association with visual function.

**Methods:**

Spectral-domain optical coherence tomography (SD-OCT) was used to evaluate both external limiting membrane (ELM) and ellipsoid zone (EZ) recovery in 40 eyes of 39 patients who underwent pars plana vitrectomy with either inverted internal limiting membrane flap technique (*n*=27) or complete ILM removal (*n*=13) to achieve MH closure. The association between ELM and EZ recovery and visual acuity was also investigated. The patients were followed up at 1 year.

**Results:**

ELM and EZ was recovered in 72% and 62% of cases, respectively, regardless of the surgical techniques 1 year after surgery. A strong positive association between the ELM and EZ recovery and the mean BCVA was found: regardless of the surgical technique, this was statistically significant at each time point (*p* < 0.05). None of the baseline variables were found to act as predictive factors for either ELM or EZ.

**Conclusion:**

The inverted ILM flap technique did not affect the MMH healing processes compared to complete ILM removal. Thus, the presence of the ILM plug did not interfere with the restoration of both ELM and EZ, which correlated with functional recovery.

## 1. Introduction

Myopic macular holes (MMHs) are one of the major causes of vision loss associated with high myopia and still represent a major cause of legal blindness worldwide [1–4]. The pathophysiological mechanism of MMHs is not completely clear. Nevertheless, several anatomical features of pathological myopia are involved, including anteroposterior, tangential traction, and retinal pigment epithelium (RPE) atrophy, as consequences of an abnormal posterior scleral wall growth leading to posterior staphyloma (PS) [5]. The surgical treatment for MMHs is still under debate. Several techniques have been attempted, including pars plana vitrectomy (PPV) with different tamponades, additional laser photocoagulation of the hole margin [6, 7], episcleral buckling of the macular area [8], and infolding the sclera [9]. Regardless, PPV associated with inner limiting membrane (ILM) peeling and gas tamponade represents the most widely chosen technique [10–16], although its results are not significantly better in comparison with the other approaches, and its MMH closure rate is inferior when compared with idiopathic MHs [17, 18]. Michalewska has reported encouraging results using the inverted internal limiting membrane (ILM) flap technique in large idiopathic MHs [19]. Subsequently, together with other authors, the same group confirmed similar positive results even for MMHs, either associated or unassociated with retinal detachment (RD) or macular schisis (MS) [20–22]. Chen and Yang compared this new technique to ILM removal for MMH-associated RDs, finding inverted ILM flap to improve anatomical and functional results [23]. More recently, our group demonstrated that inverted ILM flap was associated with better anatomical results in comparison to complete ILM removal, even in MMHs unassociated with RD [24], although no statistically significant difference in visual recovery was found between the two surgical techniques in the case of MMH closure. Although the inverted ILM flap technique has shown a higher MMH closure rate in comparison to complete ILM removal, the mechanisms by which the holes close following this procedure are still unknown. Moreover, doubts have been raised that the flap might act as a plug of fibrotic scar tissue, filling the hole. This might limit the microstructural recovery of the ellipsoid zone and interfere with the restoration of visual acuity. This hypothesis appears to be contradicted by a recent study describing reconstructive anatomical changes in foveal microstructure in both idiopathic and myopic MHs treated with inverted ILM flap [25], although the authors did not provide a comparison group. On the other hand, Iwasaki et al. evaluated the postoperative outer retinal layer recovery in eyes undergoing vitrectomy for large MH (>400 microns), finding poorer anatomical and visual results associated with inverted ILM flap compared with ILM peeling [24, 25]. These observations are in contrast with other publications and suggest a standing lack of agreement on this topic.

The aim of this study is to investigate the microstructural changes after successful MMH repair, comparing the anatomical and functional results obtained with inverted ILM flap and complete ILM removal techniques, paying special attention to factors that might have a negative functional impact.

### 1.1. Patients and Methods

This is a retrospective study based on the review of 70 consecutive patients' medical records who were affected by MMHs and who underwent surgical treatment at Sacro Cuore-Don Calabria Hospital in Verona (Italy) between 2009 and 2015. High myopia was defined as axial length >26.5 mm or refractive error >6 diopters (D). Only eyes that obtained a successful MMH closure after one surgical intervention and a minimum of twelve months follow-up were considered for the study. Only flat/closed holes, based on the classification proposed by Tornambe et al. [26], were included. Elevated/open or flat/open was considered anatomic failures. Exclusion criteria were the presence of a patchy chorioretinal atrophy involving the fovea (diagnosis based on spectral-domain optical coherence tomography (SD-OCT), showing light backscattering and the absence of outer retinal layers around the MMH), previous retinal surgery, the presence of choroidal neovascularization (CNV), diabetic retinopathy, retinal vascular occlusion, or other ocular conditions that could influence best-corrected visual acuity (BCVA), with the exception of lens opacity, inadequate imaging due to lack of quality, or impossibility to define the status of the external retinal bands secondary to long axial length and/or posterior staphyloma. Fifteen patients (14 in the ILM peeling group and 1 in the ILM flap technique) were excluded due to the lack of anatomic closure of the hole.

Patients treated before April 2013 had undergone PPV and complete ILM removal and gas tamponade, while patients treated after April 2013 underwent PPV, inverted ILM flap, and gas tamponade.

All interventions and investigations adhered to the tenets of the Declaration of Helsinki. This study was approved by the Institutional Review Board Committee of Sacro Cuore-Don Calabria Hospital. At the baseline and at every follow-up visit, a complete ophthalmic examination was performed, including BCVA measurement and SD-OCT evaluation. Axial length was also measured at the baseline (IOLMaster, Carl Zeiss Meditec, Germany). BCVA was measured using the standard Snellen eye charts. When visual acuity was evaluated with finger counting and hand motion, the measurements were assigned the equivalent Snellen acuity, namely, 20/2,000 and 20/20,000. A combined confocal scanning laser ophthalmoscope and SD-OCT (Spectralis HRA-OCT, Heidelberg Engineering GmbH, Heidelberg, Germany) was used to confirm MMH status. The size was determined by measuring the minimum diameter in OCT images. Follow-up examinations were scheduled at 1, 3, 6, and 12 months after the operation, and all patients completed the 12 months of follow-up visits. The eye-tracking dual-beam technology (TruTrack™ Active Eye Tracking Software, Heidelberg Engineering, Heidelberg, Germany) mitigates eye movement artifacts and ensures point-to-point correlations between the OCT scan and fundus images. The software also provides an “automatic real-time” (ART) function to reduce noise and increase image quality. With ART activated, multiple frames (B-scans) of the same scanning location are performed during the scanning process, and only scans with elevated ART (range 90–100) were considered. To ensure maximum scan reproducibility, the first fovea centered scan (horizontal or vertical, according to the shape of the staphyloma) acquired at the preoperative visit was set as a reference scan. To further limit misalignment errors, two expert retinal experts (MM and AA) performed and analyzed all the OCT scans. The following outer retinal features were recorded at each time point: (1) ELM integrity, (2) EZ integrity, (3) EZ gap in microns when present, and (4) the presence of MS.

### 1.2. Surgical Technique

#### 1.2.1. Complete ILM Removal

A complete vitrectomy was performed. A 23G system was used instead of smaller gauges because a longer and stiffer tip can provide better safety in highly myopic eyes. Great attention was paid to removing any remnants of the vitreous cortex after staining with triamcinolone acetonide. If present, any epiretinal membrane was peeled. Following this, the ILM was stained with a mixture of brilliant blue G and trypan blue (Membrane Dual; Dorc, The Netherlands) for about 30 seconds, and a circle approximately within the vascular arcades and centered on the macular hole was peeled off. At the end of the surgery, the eye was filled with an isovolemic mixture of sulfur hexafluoride (SF_6_). The patients were advised to maintain a face-down position for approximately eight hours a day for at least three days.

#### 1.2.2. ILM Inverted Flap Technique

The surgical procedure followed the same steps, except for the ILM removal around the MMH. The ILM peeling started about 1,000 microns from the edge of the hole and was temporarily interrupted at about 500 microns from the center. Then, most of the detached ILM was removed with the cutter. Before the air-fluid exchange, the peeling was completed up to the edge of the hole and the flap was gently positioned to cover the MMH. At the time of fluid-air exchange, low intraocular pressure and passive aspiration were used to avoid turbulence that could have caused a flap displacement. In addition, during this maneuver, the backflush needle was placed on the nasal side of the disc to generate a centripetal laminar flow to move the temporal side of the flap toward the optic disc, resulting in proper MMH roofing.

### 1.3. Statistical Analysis

Results are expressed as mean and standard deviation (SD) if the variables are continuous and as a percentage if the variables are categorical. The Shapiro–Wilk test was employed to test normality for the continuous variables. The two-sample paired *t*-test or the Wilcoxon matched-pairs signed-ranks test was used to compare the mean of continuous variables at the baseline versus the mean at subsequent follow-up. Similarly, in the subgroups, the two-sample *t*-test or the Wilcoxon rank-sum test was used to compare the mean of continuous variables for two independent groups. The linear correlation between continuous variables were assessed by the Pearson correlation coefficient (*r*). To analyze the EZ gap progression during the follow-up period, a trend test with corrections for ties was used. A stepwise logistic regression analysis at 12 months follow-up was performed to clarify whether MH diameter, MS, surgical technique, age, gender, or preoperative BCVA could have played a role in the prediction of external retina anatomical recovery. A *p* value < 0.05 was considered statistically significant. Analyses were performed using STATA version 15 (StataCorp, College Station, TX, USA).

## 2. Results

A total of 40 eyes from 39 patients met the inclusion criteria and were enrolled in the study. Twenty-seven eyes were operated on with the inverted ILM flap technique (inverted flap group), while 13 were operated on using the ILM peeling technique (peeling group). The demographic characteristics of both the peeling group and the inverted flap group are shown in Table 1.

The preoperative BCVA was slightly lower in the inverted flap group than in the peeling group at the baseline, while it was slightly higher in the same group at the end of the follow-up. Nevertheless, the difference was not statistically significant at any time point. No statistically significant differences were found between the two groups in preoperative MMH minimum diameter, axial length, and age.

**Figure 1:**
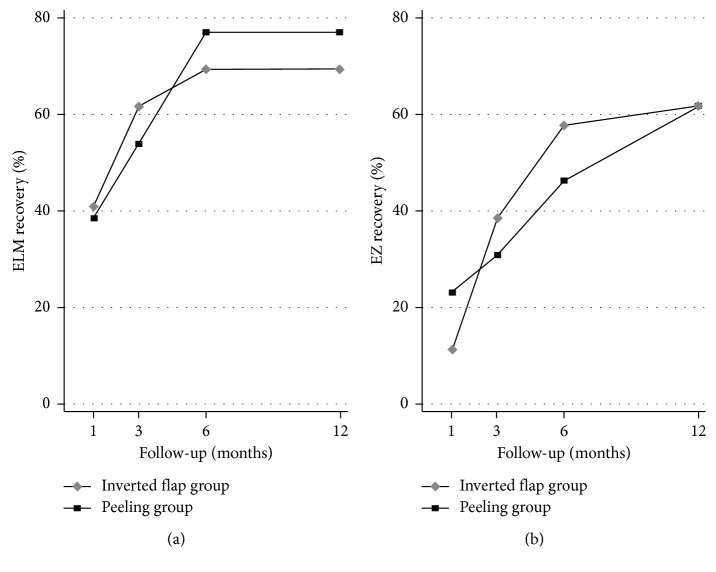
External limiting membrane (ELM) (a) and ellipsoid zone (EZ) (b) recovery rate during the follow-up in both inverted flap and peeling group. No statistically significant differences were found between the two surgical techniques at any time points.

Table 2 shows the number of eyes with a complete recovery of ELM and EZ at 1, 2, 6, and 12 months after surgery in both the peeling and inverted flap groups. The difference between the two groups was not statistically significant at any time point. Regardless of the surgical technique, ELM foveal defects decreased progressively up to 6 months, at which point the ELM recovered in 72% of the examined subjects. No further improvements were observed in either group at 12 months. On the other hand, EZ recovery progressed steadily throughout the whole follow-up period, reaching completion in 62% of the investigated subjects (Figure 1).

**Figure 2:**
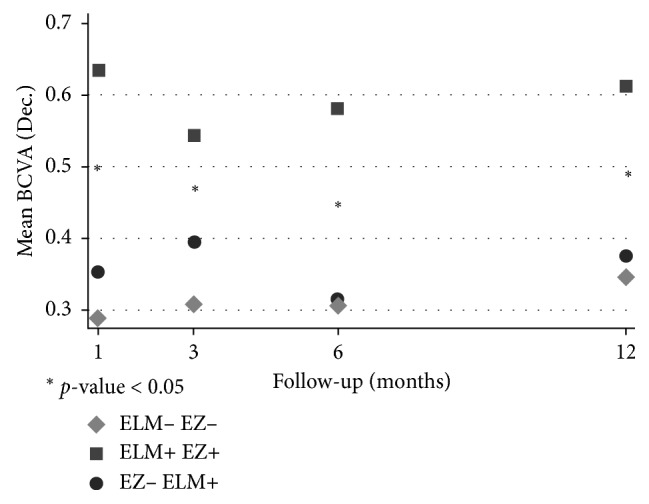
Association between best-corrected visual acuity (BCVA) and external limiting membrane (ELM) and ellipsoid zone (EZ) recovery during the follow-up in the whole sample. Regardless of the surgical technique, a statistically significant positive association between BCVA and ELM/EZ restoration was found at each time point. In Table 2, the number of cases with ELM and EZ recovery is shown for each time point. Asterisks indicate a statistically significant difference between the two contiguous points (*p* < 0.05). ELM− EZ− = neither ELM nor EZ recovery; ELM+ EZ− = ELM recovery; ELM+ EZ+ = ELM and EZ recovery.

We also investigated the association between functional and morphological parameters, finding a strong positive association between the ELM and EZ recovery and the mean BCVA. Regardless of the surgical technique, this was statistically significant at each time point (*p* < 0.05) (Figure 2).

The subfoveal EZ gap was also measured at each time point. A statistically significant decreasing trend was found when the whole sample was studied (*p* value < 0.0001) and also when the analysis stratified the two surgical techniques (peeling group *p* value = 0.014; inverted flap group *p* value = 0.005).

The role of the preoperative variables included in Table 1 in predicting ELM and EZ restoration was investigated. According to the stepwise logistic regression analysis at 12 months, none of the preoperative variables were significantly predictive factors of either ELM and EZ recovery or EZ gap reduction.

**Figure 3:**
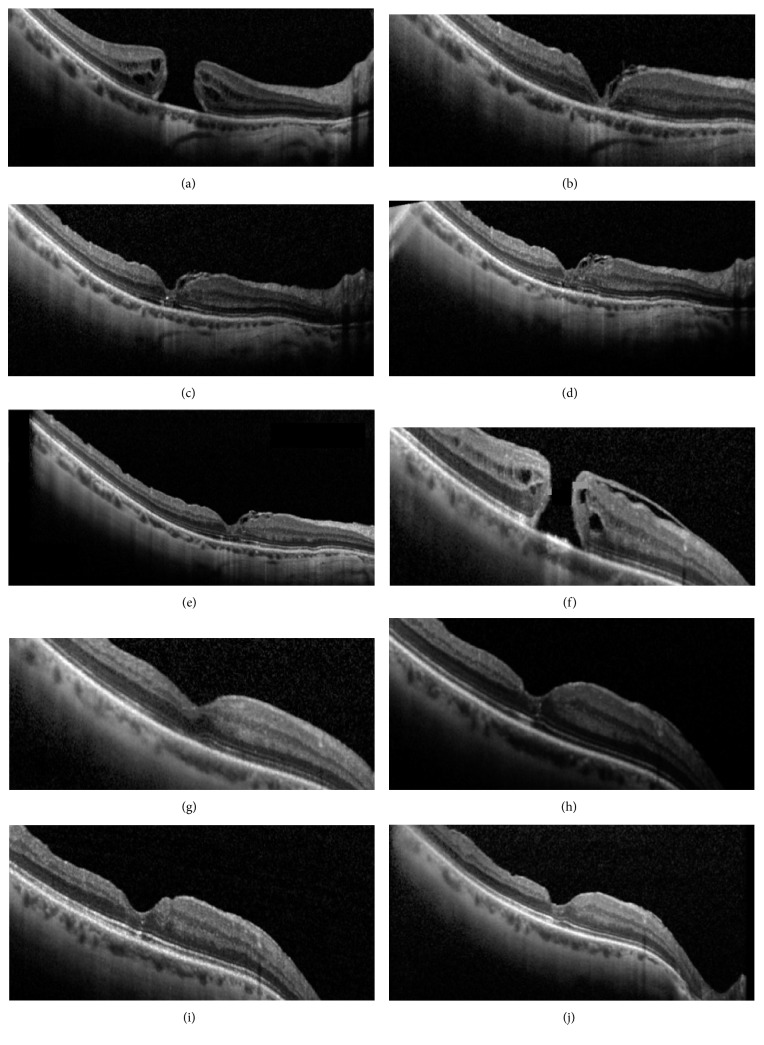
Follow-up of 2 cases of myopic macular holes (MMHs), namely, operated on using inverted internal limiting membrane (ILM) flap technique (a–e) and with complete ILM removal (f–j). Regardless of the surgical technique, ELM and EZ recovered progressively, even if ELM recovered first. In the inverted ILM flap case, a progressive reabsorption of the ILM plug was also noticed. (a, f) Preoperative. (b, g) 1 month after surgery. (c, h) 3 months after surgery. (d, i) 6 months after surgery. (e, j) 12 months after surgery.

Figure 3 reports two cases belonging to the inverted ILM flap group and peeling group, respectively. The baseline characteristics of the two eyes were similar in terms of myopia grade and MMH diameter. During the follow-up, ELM recovered before EZ in both eyes. In the inverted ILM flap group, a progressive reabsorption of the hyperreflective material corresponding to the ILM flap was also noticed. One year after surgery, ELM and EZ recovery was completed and the two cases appeared to be extremely similar.

## 3. Discussion

The present study performed a comparison of anatomical and functional results obtained with inverted ILM flap and complete ILM removal techniques for MMH treatment. The results showed no significant differences in either the microstructural changes occurring after successful MMH repair with the two techniques or in postoperative BCVA.

The MMH closure rate has varied widely amongst authors [27, 28], and a correlation between axial length and MMH closure has never been demonstrated [20, 23]. The majority of OCT-based studies have confirmed the clinical suspicion that the closure rate of myopic MHs was much lower than in idiopathic cases and ranged from 60 to 90% [17, 18]. This discrepancy is likely to be related to the limited elasticity of the retina, which should be stretched to cover the staphyloma [1]. Therefore, an elevated/open or flat/open MH shape has been frequently reported after surgery for MMH after vitrectomy and ILM peeling [29–32].

Michalewska first demonstrated the effectiveness of the inverted ILM flap technique, both in large idiopathic MHs (>400 microns) [19] and in MMHs [21]. The inverted ILM flap technique was shown to be effective in improving both visual acuity and the MH closure rate. Other authors presented similar results in MMHs [10, 22], even when associated with RD [10]. More recently, an inverted ILM flap and complete ILM removal were compared for the treatment of MMHs associated or unassociated with RD. The inverted ILM flap was confirmed as associated with better anatomic results [23, 24]. Nevertheless, its exact mechanism of action is not completely clear and some ophthalmologists are skeptical about this technique as they assume that the presence of an ILM plug inside the hole could affect the functional outcome. In other words, the inverted ILM flap technique might improve the closure rate of MMHs, yet jeopardize improvement in visual acuity.

In this study, we found no statistically significant difference in postoperative BCVA between the eyes treated with complete ILM peeling and those that received an ILM flap. The absence of a significant functional difference between the two groups suggests that the presence of the ILM plug did not affect visual recovery in our series. However, very little has been published thus far about an inverted ILM flap possibly interfering with the MH healing process. Very recently, Iwasaki proposed evidence in opposition to our data, with a retrospective study on 24 eyes undergoing either ILM peeling (*n*=10) or inverted ILM flap (*n*=14) for large MH (<400 microns). The authors reported both a significantly lower recovery rate and ELM recovery time in the inverted ILM flap group compared to the ILM peeling group [33]. However, there is potential bias in this study. First, the number of cases in the 2 groups is rather low (10 and 14) and the authors do not indicate the technique selection criteria. Second, the mean hole diameter in the ILM flap group was more than 100 microns higher than in the ILM peeling group and likely did not reach full statistical significance (*p*=0.053) only because of the low numbers of eyes included in the study. Third, a minimum follow-up of 6 months is quite short to exhaustively assess the recovery of the outer retina layers. Moreover, the sample is quite heterogeneous as it includes two highly myopic cases and an eye with inactive diabetic retinopathy. Nevertheless, these opposing hypotheses confirm that the mechanism of the inverted ILM flap needs further investigation, even if there is enough evidence that it promotes MH closure, especially in the case of large diameter and myopic eyes.

The dynamic and progressive repair of the outer retinal layer after vitrectomy and ILM peeling for idiopathic MH was described in detail by Bottoni et al. [34]. ELM restoration was reported as complete within 1–3 months, while EZ restoration could take up to 12 months to occur. The authors observed that the recovery of ELM, EZ, and the outer nuclear layer (ONL) took place months after the MH closure and were associated with visual acuity improvement. Our series has demonstrated a similar behavior, as shown in Figure 2. A statistically significant association between ELM/EZ integrity and BCVA was observed at each time point. The positive relationship between ELM integrity and BCVA has already been demonstrated in other diseases, like macular degeneration [35, 36] and diabetic macular edema [37, 38].

Interestingly, we found that the timing and restoration rate of the ELM and EZ did not differ significantly in the inverted flap group compared to the ILM peeling group (Figure 1). This represents a remarkable observation as it indicates that the ILM plug does not interfere with the physiological healing process described after ILM peeling [34].

As shown in Figure 3, the OCT scans revealed the presence of hyperreflective material in close contact with RPE 1 month after surgery. This is likely related to the ILM flap present inside the hole. During the follow-up, a displacement of this material toward the inner retinal layers occurred, and at the same time, the ELM became detectable. This observation may support the ILM flap's role as a scaffold for retinal layer repair after surgery for both idiopathic and myopic MHs [19, 39, 40]. Moreover, we hypothesized that the ILM, creating a separation between the vitreous cavity and the intraretinal environment, can promote the restoration of a favorable homeostatic condition for hole closure [41].

Hayashi and Kuriyama [25] described the microstructural changes after vitrectomy and ILM flap technique in patients that suffered from MMHs with or without RD, but unfortunately, they did not provide a control group with complete ILM peeling. Despite the same surgical technique, they found better outer retinal layer restoration in eyes without RD, probably because the outer retina detachment results in photoreceptor damage that cannot be recovered even after MMH closure. In addition, it is likely that a RD induced by a MMH occurs more frequently when extended chorioretinal atrophy and a deep staphyloma are present. Therefore, microstructural recovery and functional improvement are less likely in light of the underlying pathology.

Our statistical analysis did not indicate any predictive factor for the microstructural outcomes of surgery. The MMH preoperative diameter, axial length, and surgical technique did not influence MMH closure processes secondary to surgery. Nevertheless, we only analyzed eyes that reached a flat, closed appearance on OCT. It should be reiterated that, in previously reported data, the ILM flap technique was found to have a statistically significant higher rate of MMH closure than the ILM peeling technique [24]. We can therefore conclude that, based on our experience, the ILM flap technique is preferable as it improves the anatomic outcome and has no negative implication for functional results.

A free flap of ILM transplantation has been proposed for large MHs after failed surgeries with ILM removal [42]. This is our choice for both idiopathic and myopic refractory MHs that had already undergone ILM peeling around the hole. Recently, autologous neurosensory retinal free flap transplantation has been suggested for repairing refractory MMHs [43]. The authors speculated the retinal free flap may improve both BCVA and retinal sensitivity after surgery. We think that it is unlikely that a retinal free flap could conduct a signal to the optic nerve as creating the flap interrupts the inner retinal circulation. Thus, the retinal free flap is likely to act as a tissue to effectively separate the subretinal space from the vitreous cavity, promoting spontaneous MH healing processes. The possibility that the transplanted retinal flap may represent a source of stem cells that can migrate into the native retina and promote outer retinal layer repair is speculative and needs further investigation. A retinal free flap should probably be considered only when residual ILM cannot be found to cover the MMH. For this reason, Wong and Steel [44] suggest not overextending the ILM peeling to preserve ILM for creating a free flap if the MMH failed to close.

In conclusion, in our series of successfully closed MMHs, we found that the inverted ILM flap technique did not affect the MMH healing processes compared to complete ILM removal. Thus, the presence of the ILM plug did not interfere with the restoration of both ELM and EZ, which correlated with functional recovery. A significant limitation of this study is the lack of randomization due to its retrospective nature. However, previous studies demonstrated that the inverted ILM flap technique resulted in a higher closure rate of MMHs in comparison to complete ILM removal [24]; therefore, we believe that it should be considered as the technique of choice for the treatment of MMHs. Nevertheless, this technique's exact mechanism of action should be fully clarified to understand its real potential.

## Figures and Tables

**Table 1:** Baseline demographics factors for patients affected by myopic macular hole (MMH).

	Inverted ILM flap (*n*=27)	ILM peeling (*n*=13)	*p* value	Total (*n*=40)
Gender (M : F)	7 : 20	3 : 10	—	10 : 30
Age (years), mean (SD)	57.8 (10.0)	62.9 (13.3)	0.1179	59.5 (11.3)
Eye (right-left)	14 : 13	6 : 7	—	20 : 20
BCVA baseline decimal, mean (SD)	0.24 (0.11)	0.28 (0.13)	0.1644	0.25 (0.12)
Axial length (mm), mean (SD)	29.3 (2.9)	31.0 (3.0)	0.1340	29.7 (3.0)
MMH minimum diameter in *µ*m, mean (SD)	440 (191)	368 *µ*m (179)	0.1258	416 (188)
Lens				
Aphakic	1 (4%)	0 (0%)		1 (2.5%)
Pseudophakic before Vx	11 (41%)	9 (69%)		20 (50%)
Phacoemulsification during Vx	9 (33%)	3 (23%)		12 (30%)
Phacoemulsification after Vx	1 (4%)	1 (8%)		2 (5%)
Cataract	5 (18%)	0 (0%)		5 (12.5%)

**Table 2:** Eyes demonstrating complete restoration of external limiting membrane (ELM) and ellipsoid zone (EZ) integrity at different time points.

	1 month	3 months	6 months	1 year
Whole sample (*n*=40)				
ELM, *n* (%)	16 (40)	23 (59)	28 (72)	28 (72)
EZ, *n* (%)	6 (15)	14 (36)	21 (54)	24 (62)
Inverted flap (*n*=27)				
ELM, *n* (%)	11 (41)	16 (62)	18 (69)	18 (69)
EZ, *n* (%)	3 (11)	10 (38)	15 (58)	16 (62)
ILM peeling (*n*=13)				
ELM, *n* (%)	5 (39)	7 (54)	10 (77)	10 (77)
EZ, *n* (%)	3 (23)	4 (31)	6 (46)	8 (62)
ELM *p* values (inverted vs peeling)	0.4519	0.3147	0.2996	0.2996
EZ *p* values (inverted vs peeling)	0.1591	0.3326	0.2378	0.5000

## Data Availability

The data used to support the findings of this study are available from the corresponding author upon request.
